# Does Head Orientation Influence 3D Facial Imaging? A Study on Accuracy and Precision of Stereophotogrammetric Acquisition

**DOI:** 10.3390/ijerph18084276

**Published:** 2021-04-17

**Authors:** Giuditta Battistoni, Diana Cassi, Marisabel Magnifico, Giuseppe Pedrazzi, Marco Di Blasio, Benedetta Vaienti, Alberto Di Blasio

**Affiliations:** 1 Section of Orthodontics, Dental School, Department of Medicine and Surgery, University of Parma, 43126 Parma, Italy; marisabel.magnifico@libero.it (M.M.); marco.diblasio@studenti.unipr.it (M.D.B.); benedetta.vaienti@studenti.unipr.it (B.V.); alberto.diblasio@unipr.it (A.D.B.); 2 Department of Surgical, Medical, Dental and Morphological Science with Interest in Transplant Oncological and Regenerative Medicine, University of Modena and Reggio Emilia, 41124 Modena, Italy; dianacassi3@gmail.com; 3 Department of Medicine and Surgery, Unit of Neuroscience, Interdepartmental Centre of Robust Statistics (Ro.S.A.), University of Parma, 43126 Parma, Italy; pedrazzi@unipr.it

**Keywords:** stereophotogrammetry, head position, orthodontics, 3D imaging, anthropometry, dentistry craniofacial morphology, non-invasive imaging

## Abstract

This study investigates the reliability and precision of anthropometric measurements collected from 3D images and acquired under different conditions of head rotation. Various sources of error were examined, and the equivalence between craniofacial data generated from alternative head positions was assessed. 3D captures of a mannequin head were obtained with a stereophotogrammetric system (Face Shape 3D MaxiLine). Image acquisition was performed with no rotations and with various pitch, roll, and yaw angulations. On 3D images, 14 linear distances were measured. Various indices were used to quantify error magnitude, among them the acquisition error, the mean and the maximum intra- and inter-operator measurement error, repeatability and reproducibility error, the standard deviation, and the standard error of errors. Two one-sided tests (TOST) were performed to assess the equivalence between measurements recorded in different head angulations. The maximum intra-operator error was very low (0.336 mm), closely followed by the acquisition error (0.496 mm). The maximum inter-operator error was 0.532 mm, and the highest degree of error was found in reproducibility (0.890 mm). Anthropometric measurements from alternative acquisition conditions resulted in significantly equivalent TOST, with the exception of Zygion (l)–Tragion (l) and Cheek (l)–Tragion (l) distances measured with pitch angulation compared to no rotation position. Face Shape 3D Maxiline has sufficient accuracy for orthodontic and surgical use. Precision was not altered by head orientation, making the acquisition simpler and not constrained to a critical precision as in 2D photographs.

## 1. Introduction

The quantitative analysis of the human face has always received large attention from both scientists and artists [1]. Qualitative analysis of the face is a daily, often unconscious, process. Facial appearance allows personal identification, communication, and interaction with the environment, and it gives information about the individual’s health state. Anthropometric evaluation is carried out by several medical specialists using techniques that require accuracy and precision [2]. In this way, aesthetic and maxillofacial surgeons, otolaryngologists, dentists, oral surgeons, and orthodontists can all document clinical cases and compare different images of the same patient (e.g., pre-treatment and post-treatment) [3]. An objective, accurate, and reliable system for quantifying the soft tissues of the face in three dimensions and in color is still being studied [4].

Anthropometric analysis of soft tissues is an integral part of orthodontic diagnosis along with therapeutic planning, and two-dimensional (2D) photography has been, for years, one of the main devices for the analysis of facial measurements. Despite this, such a technique carries considerable limitations. It can reproduce reality but only in two dimensions, leaving out valuable information about the depth and the transversal dimension of a face. Moreover, in 2D the position of the patient inside the framework of the image is critical. Any error in the vertical, horizontal, or rotational positioning of the face during the shot creates distortions, making images practically useless. A 3D photograph, on the contrary, can be shot even in the presence of less than ideal positioning, which can then be easily corrected on the computer. The 3D image, therefore, is not only a general improvement of 2D, but a means that provides a completely new concept of the image, by being able to perform measurements and overlaps and, thus, making objective what was previously only a clinical impression. In addition, stereophotogrammetry provides an accurate assessment of the aesthetic outcome of the therapy, which, being not invasive, can also be repeated with frequent surveys over time. Moreover, even if clinicians and researchers are still discussing how it may be possible to reach a reproducible natural head position (NHP), the smoothest variations on the positioning of the patient’s head during a 2D photography session could unavoidably alter results within a single assessment or time-to-time comparisons. 

Interest in overcoming the limitations of direct measurements and of 2D photogrammetry has led to the development of numerous non-invasive methods for capturing and quantifying craniofacial surface morphology [5]. 

Today, we have sophisticated digital scanning devices that complete our evaluations with third-dimension (3D) data, and even more recently the fourth dimension (4D), that includes movement [6,7,8]. In the literature, various 3D imaging systems of soft facial tissues are described; currently, the gold standard is represented by three-dimensional stereophotogrammetry [9,10]. Each new stereophotogrammetric system requires a validation process that establishes accuracy and precision before clinical use by identifying possible grinding and extrinsic errors to the system. 

Chung et al. [11] give an overview of the three-dimensional scanning device types available. Despite the huge amount of literature about the new three-dimensional system, a clear and objective evaluation of accuracy and reliability under different circumstances is missing in many studies. Verifying any in vivo hypothesis necessarily presupposes a previous validation of the system in vitro, in terms of technical validation and knowledge of system errors in different acquisition conditions. 

The goals of this study are (1) to validate the present digital 3D photogrammetric device in terms of measurement error; (2) to compare craniofacial measurements obtained from 3D images which were generated from alternative captures of a mannequin head with different degree of yaw, pitch, and roll rotations. The hypothesis is that anthropometric measures recorded in different conditions of head orientation are equivalent to each other. 

## 2. Materials and Methods 

### 2.1. Polishape Technology

The scanner we used was Face Shape 3D Maxi Line, developed by Polishape 3D Srl (Bari, Italy). This photogrammetric system counted six Canon EOS 1100D (12.2 megapixels, lenses focal length: 50 mm) reflex cameras, each one fixed on rigid support with a specific inclination. Two lateral and external professional flashes were used to minimize any possible external light distortions and to obtain uniform light on the surface of the object. 

### 2.2. Object and Data Acquisition

After the required calibration procedure, the subject could be shot and the corresponding 3D image created by a specific software by a 3D rendering function; in this study, we used Viewbox 0.4^®^ (dHAL Software, Kifissia; Greece).

**Figure 1:**
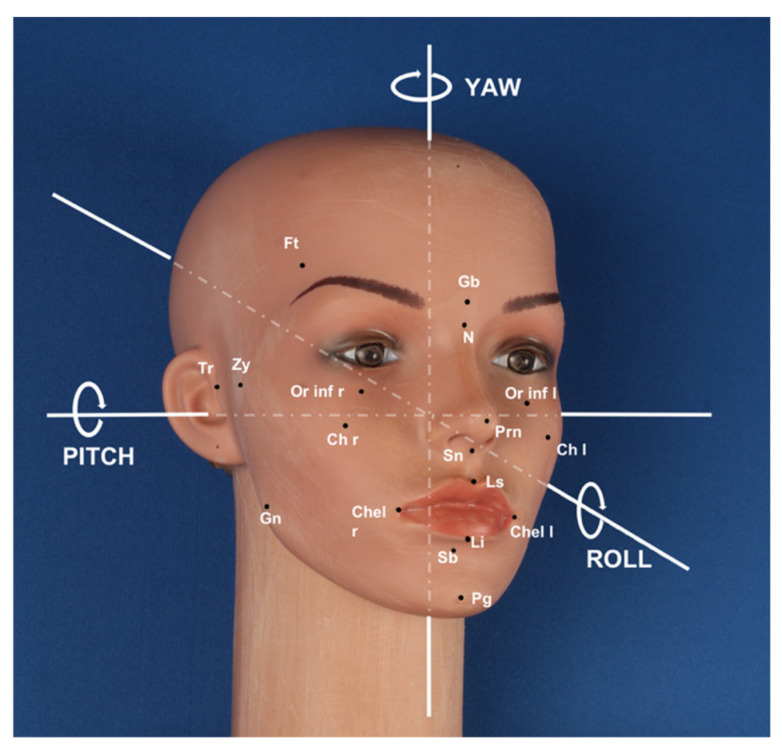
Dummy: anthropometric points and illustration of movements. A three-quarter representation of the mannequin’s head with anthropometric points marked in black. Additionally, the pitch, roll, and yaw movements are expressed with white lines and arrows that simulate the movement.

We used a mannequin head as a subject, which is ideal for in vitro experimentation due to immobility and absence of facial mimicry. To improve image acquisition, the texture of the dummy was faded and opaque to reduce light reflection, and, as suggested by the literature data [12], the following 22 anthropometric points were marked with an eyeliner (Figure 1):

- 8 median: Glabella (Gb), Nasion (N), Pronasale (Prn), Subnasale (Sn), Labiale superius (Ls), Labiale inferius (Li), Sublabiale (Sl), and Pogonion (Pg);
- 7 bilateral: Frontotemporalis (Ft r/l), Zygion (Zy r/l), Tragion (Tr r/l), Gonion (Gn r/l), Cheek (Ch r/l), Cheilion (Chel r/l), and Orbitale inferius (Or inf r/l).

The dummy was placed on external support equipped with a graduated scale that allowed the operator to orientate the dummy on the three planes of the space with extreme control on specific angular rotational values during the pitch, roll, and yaw movements (Figure 1).

To investigate the accuracy and precision of the system, a series of captures was taken with the mannequin head with no rotation (reference position) and with various pitch, roll, and yaw angulations. The protocol of image acquisition is reported in Table 1.

Next, all shots were processed, and the anthropometric points previously marked on the dummy were also marked on the three-dimensional reconstructions obtained. Their *x*, *y*, and *z* coordinates were collected from the software and saved in an Excel spreadsheet. 

**Figure 2:**
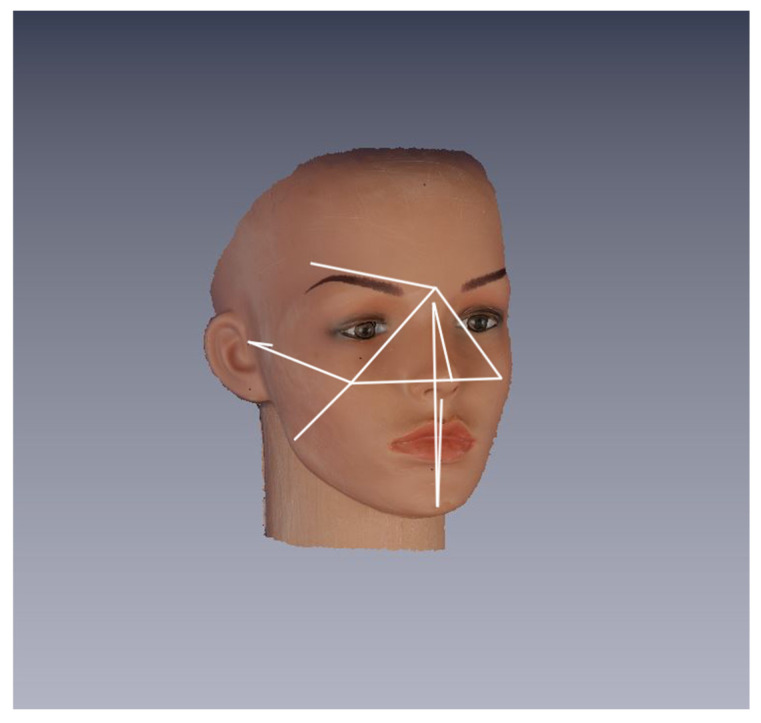
Dummy: linear distances between anthropometric points. A three-quarter projection of the dummy’s head with a representation of linear distances measured on the 3D image between pre-labeled landmarks.

The following 14 linear distances have been calculated (Figure 2):

- 4 median: Chel(r)-Chel(l), N-Prn, Sn-Pg, and N-Pg;
- 5 bilateral: Glab-Ch(r), Glab-Ch(l), Glab-Ft(r), Glab-Ft(l), Zy(l)-Tr(l), Zy(r)-Tr(r), Ch(r)-Tr(r), Ch(l)-Tr(l), Ch(r)-Gn(r), and Ch(l)-Gn(l).

The 14 measurements were chosen to cover various facial regions, having different size and orientation on the transverse, frontal, and sagittal plane. 

### 2.3. Data Processing and Operational Definitions

The Euclidean distance between two landmarks has been calculated as the square root of the sum of the differences in the three dimensions of the space, as indicated in the following formula: $d=\sqrt{\Delta x^{2}+\Delta y^{2}+\Delta z^{2}}$, an analog to the target registration error (TRE) described in several articles [13,14]. 

Errors may be introduced during imaging acquisition, placement of landmarks on the images, or calculation of distances. To estimate the relative contributions of these sources of errors, the precision of the system was investigated in terms of repeatability (same team, same experimental set-up) and reproducibility (different team, different experimental set-up). Notably, repeatability included intra-operator, inter-operator, and acquisition errors.

All the investigators were orthodontists, with at least 20 years of clinical experience in recognizing anthropometric points.

To assess intra-operator error, the same investigator repeated (10 times) the placement of anthropometric landmarks and measurements of linear distances on the same 3D reconstruction taken in the reference position. All parameters were measured again by a second investigator on the previous acquisition and compared (inter-operator error). Acquisition error was assessed by measuring, using the same operator, the selected parameters of 5 different 3D image captures in reference position. To investigate reproducibility error, we compared measurements performed by the two operators on different acquisitions. To avoid recall bias, a minimum of 24 h was allowed to elapse between measurement sessions.

To assess how the head position might affect the accuracy of anthropometric measurements, each set of linear measurements of yaw, roll, and pitch was compared with data obtained from the reference position, and the mean value of the linear measurements derived from each set of 3D images taken with yaw, roll, and pitch angles was compared to the mean of those obtained in the reference position. 

### 2.4. Data Analysis

Statistical analysis was performed using various software, among them the open-source statistical software Jamovi [15], which is based on the widespread open-source statistical system “R”, the free epidemiological software Winpepi [16], and Microsoft Excel. 

To quantify measurement error magnitude, the following measurement error indices were calculated: mean error, maximum error, standard deviation (of errors), standard error (of errors), and coefficient of variation (CV).

To demonstrate the equivalence between measurements obtained in alternative head positions, the mean difference between repeated measures was calculated, and the Westlake–Schuirmann two one-sided test (TOST) was used [17,18]. The mean equivalence of two sets of measurements was defined as a difference not higher than the maximum error of the system, which had been previously calculated in the reference position. The null hypothesis for equivalence was that there was a substantial difference (greater than the maximum error) between the measurements performed in different conditions of head orientation. In the case of rejection of the null hypothesis, equivalence can be assumed. It must be noted that the null hypothesis for equivalence test is just the opposite of that conventionally used in superiority tests, where rejection of the null states that a difference exists. To prove equivalence, the test must reject the hypothesis of difference [17,18]. The test was repeated for each set of measurements generated from the captures of the dummy with yaw, pitch, and roll angulations compared to those with no rotation. Results were considered statistically significant for a p-value less than 5% (*p* < 0.05). Bonferroni’s correction was applied for multiple comparisons.

## 3. Results

### 3.1. Intra-Operator Error 

The mean of standard errors was 0.041 mm, with a minimum value of 0.023 mm in the Glabella–Frontotemporal (l) measurement and a maximum value of 0.081 mm in the Nasion–Pogonion measurement; such a measurement also reached the highest maximum error (0.336 mm). 

### 3.2. Inter-Operator Error

The inter-operator standard error was higher than the intra-operator value with a mean value of 0.064 mm, a minimum value of 0.04 mm in the Chelion–Gonion (r) measurement, and a maximum value of 0.111 mm in the Nasion–Pogonion measurement, whose maximum error was found to be 0.532 mm. 

### 3.3. Acquisition Error 

The mean standard error of acquisition was 0.103 mm with a minimum value of 0.066 mm for Glabella–Frontotemporale (r), and a maximum value of 0.177 mm for Zygion (r)– Tragion (r). The highest maximum error was found in the Cheek (l)–Tragion (l) measurement of 0.496 mm. 

### 3.4. Reproducibility Error 

The mean standard error was 0.086 mm with a distribution of values between 0.032 mm for Glabella–Frontotemporale (r) and 0.164 mm for Cheek (l)–Tragion (l). The latter is also characterized by the largest value of the maximum recorded error of 0.890 mm. 

**Figure 3:**
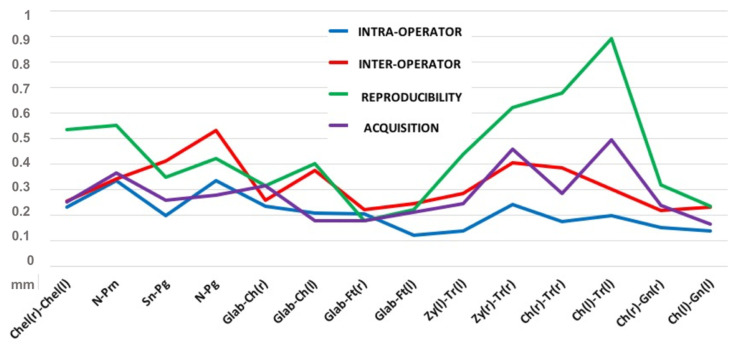
Maximum error distribution (mm).

The error magnitude statistics are reported in Table 2, and the maximum error distribution is represented in Figure 3. 

**Figure 4:**
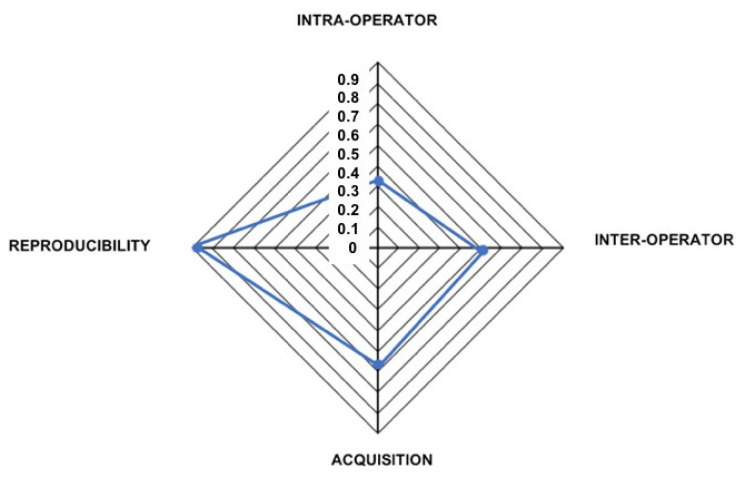
Total error components. This figure reports the impact of various sources of errors: the maximum intra-operator error is 0.336 mm, the maximum acquisition error is 0.496 mm, the maximum inter-operator error is 0.532 mm, and the maximum reproducibility error is 0.890 mm.

Figure 4 reports the impact of various sources of errors. As expected, the maximum intra-operator error was very low (0.336 mm) as it contained only one variable: the operator itself. It was followed by the acquisition error (0.496 mm), which resulted from repeated captures under the same condition, and is intrinsic to the imaging device. Adding the variable of a second operator (inter-operator error), the maximum error increased to the value of 0.532 mm, thus suggesting that the “operator” variable played a greater role in increasing the variability of the results. 

The higher degree of maximum error was found in reproducibility (0.890 mm), which combined both the error due to digitization and the imaging system.

### 3.5. Error Analysis and Rotations Equivalence

The statistical equivalence between anthropometric measurements coming from the alternative acquisition conditions was calculated considering an equivalence limit of 0.89 mm, which represented the maximum error of the measurement system at the reference position.

The mean difference between linear measures and equivalence data is reported in Table 3.

Yaw versus reference position: the two sets of measurements were found to be statistically equivalent (test of equivalence: *p* <0.01). The highest mean difference was 0.422 mm for the Cheek (l)–Tragion (l) measurement.

Roll versus reference position: the two series of measurements were found to be statistically equivalent (test of equivalence: *p* <0.01). The maximum difference between the averages was 0.543 mm calculated for Zygion (l)–Tragion (l).

Pitch versus reference position: the two sets of measurements were statistically equivalent (test of equivalence: *p* < 0.05) with the exception of two distances, Zygion (l)–Tragion (l) (*p* = 0.510) and Cheek (l)–Tragion (l) (*p* = 0.166). The maximum difference between the averages was 0.814 mm calculated for Zygion (l)–Tragion (l).

## 4. Discussion

The goal of the present study was to analyze a new six-camera stereophotogrammetry system and to evaluate the accuracy and the reliability of craniofacial measurements obtained from 3D surface captures with different degrees of head orientation. In particular, the analysis focused on searching for errors, whether they were specific to the system or related to the operator.

To the best of our knowledge, no studies have been performed to investigate the accuracy of the present stereophotogrammetric system in a clinical setting, and there are no studies on measures variations induced by head rotations in the three planes of space.

A previous study [19] has demonstrated that facial landmarks do not have the same reproducibility dividing them into highly, moderately, and poorly reproducible landmarks. Examples such as Zygion, Gonion, and Tragion have been shown to have poor reproducibility.

In this study, the highest maximum error was recorded in the measurements involving the Tragion. We can assume that the removal of these parts, reducing the measurements onto the facial oval, would improve the precision. If we exclude measurements made in the lateral regions, the maximum error drops considerably (0.535 vs. 0.89 mm).

Intra-operator maximum error was very low (0.334 mm), and acquisition error and inter-operator error were very similar (0.496 mm/0.532 mm). As expected, the maximum error of reproducibility was the highest (0.89 mm). Reproducibility refers to the variation of measurements made under changing conditions, which in the present experiment were due to measurements being made by two operators on acquisitions with different head angulations; thus, such a value combines various types of errors owing both to digitization and to the imaging system itself. Lateral regions, along with interoperability variability, were sources of greater acquisition and reproducibility errors, especially in measurements involving the Tragion area.

In the context of medical facial treatment, a patient’s photographs represent an extremely important datum both for the follow-up and simple clinical documentation. So far, in 2D photographs it has always been the practice to search the NHP of the patient’s head as a reference position. Despite that, among the scientific community, there is still discussion on how to reach the NHP and whether it can be reproducible.

Cassi et al. [20], in a recent review, focused on techniques to establish the NHP, and how to transfer it to the cephalostat, together with an overview of the three-dimensional recording methods recently introduced into clinical practice. Several studies have successfully measured the reproducibility and stability of the NHP, both in a short and long time-lapse [21,22,23].

On the other hand, although the NHP has less variability than intracranial reference lines, it is also influenced by balance, vision, and proprioception from joints and muscle involved in maintaining erect posture. Therefore, it depends on the subject’s neuromuscular condition as well, and it may be difficult to obtain in some patients, especially children, and subjects with neuromuscular disorders, vertebral column deformity, and alterations in eye muscles balancing [24].

In the literature, some protocols for obtaining the NHP might influence reproducibility, and there is also some evidence that the success might depend on the operator [25]. To some other authors [26], the perception of correct anatomical alignment changed considerably with time. They say that different observers disagreed on the correct anatomical alignment, and the agreement among multiple observers was bad for pitch, moderate for yaw, and good for the roll. Therefore, even if the NHP was perfectly repeatable, the problem of the correct tracking of the camera would remain. The evidence that a change in the relative position of the face/camera system in pitch/roll/yaw does not compromise the result (at least in clinical terms) is a relief, not only from the “NHP problem” but also from the problem of the alignment of the detection system of the image.

In light of this, since the facial scanning system is continuously being developed as a valid alternative to 2D photography, we wanted to investigate if head rotations on the three spatial planes could represent any critical aspects in terms of reproducibility and precision, with notable implications on the success of the results.

We can accordingly conclude that the position of the dummy does not influence the precision, accuracy, and repeatability of anthropometric measurements, at least within 16 degrees on right and left for yaw and roll, and within 11 degrees upward and downward for pitch.

These results agree with data from studies conducted with other stereophotogrammetric systems. In fact, Lubber et al. stated that by progressively changing the spatial orientation of a dummy (by rotating and translating its head from a neutral position), the mean error of measurements on the corresponding 3D image remained very low and steady within the central range of movement (TRE: 0.195 mm), which then showed very little increases along with large spatial variations of the dummy [27]. Moreover, other authors assess that images captured by a stereophotogrammetric device are highly repeatable. In their study, the same authors have found the error associated with the placement of landmarks on the 3D images to be sub-millimeter, therefore irrelevant enough to be able to assert that landmark digitalization can be acquired with a high degree of precision using this technology [28]. Ayoub et al. [29] identified operator error to be accurate within 0.2 mm, and the average discrepancy of point location for three operators involved was 0.79 mm.

## 5. Conclusions

In conclusion, we can state that the Face Shape 3D Maxi Line has sufficient accuracy for orthodontic and surgical use, especially in the median areas of the face. Despite the presence of areas of non-equivalence (lateral areas), the differences are clinically acceptable. Considering the magnitude of the intra-operator and inter-operator errors, which represent a significant proportion of the total error, we might suppose also that acting on them and the learning curve might reduce the system error.

Based on the results, we might assert that such precision is not altered by the rotation of the head on three planes of space, making the acquisition process even simpler and less constrained to a predetermined position, which is not always easy to obtain with all patients. This improves the comparison of acquisitions obtained at different times and conditions, facilitating the clinician during long-term treatment.

### Strengths and Limitations

The mannequin head represented an ideal object, as it did not move or perform facial expressions. This allowed it to reach a very high degree of precision, without the influence of human variability on the photographed subject. On the other hand, the estimates of precision and accuracy might be inflated by the experimental setting, and the lack of a human sample is a limitation to the study, namely not being able to assess the effect of the stretching of the soft tissues in the precision of the measurement.

Further studies are needed to confirm the results obtained in vitro by repeating the study in vivo. Living subjects, as opposed to inanimate mannequin heads, may be affected by motion artefacts such as breathing or swallowing, and thickness and soft tissue characteristics, also related to different age ranges, might influence the measurement error of 3D surface imaging systems.

## Figures and Tables

**Table 1:** The protocol of image acquisition reported the 58 captures that were taken as follows: 10 captures with the mannequin head with no rotation (0 degrees), defined as reference position; 16 captures with the mannequin head rotated at different degrees of yaw, respectively 1, 2, 3, 4, 5, 8, 12, and 16 degrees, both on right and left; 16 captures with the mannequin head rotated at different degrees of rolls, respectively 1, 2, 3, 4, 5, 7, 9, and 11 degrees, both on right and left; 16 captures with the mannequin head rotated at different degrees of pitch, respectively 1, 2, 3, 4, 5, 7, 9, and 11 degrees, both upward and downward.

Head Position	Rotation	Number of Captures
REFERENCE POSITION	None	10
YAW	1, 2, 3, 4, 5, 8, 12, and 16 degrees (right) 1, 2, 3, 4, 5, 8, 12, and 16 degrees (left)	16
ROLL	1, 2, 3, 4, 5, 8, 12, and 16 degrees (right) 1, 2, 3, 4, 5, 8, 12, and 16 degrees (left)	16
PITCH	1, 2, 3, 4, 5, 7, 9, and 11 degrees (upward) 1, 2, 3, 4, 5, 7, 9, and 11 degrees (downward)	16

**Table 2:** Error magnitude statistics: the table shows the descriptive statistics used to quantify measurement error magnitude. Maximum error, standard deviation, standard error, and coefficient of variation were calculated for each distance between landmarks in investigating intra-operator, inter-operator, acquisition, and reproducibility error. All measures are expressed as mm.

Measurements	Error Indices	Intra-Operator (mm)	Inter-Operator (mm)	Acquisition (mm)	Reproducibility (mm)
Chel(r)-Chel(l)	maximum error	0.231	0.257	0.253	0.535
	standard error	0.042	0.052	0.084	0.106
	standard deviation	0.133	0.165	0.187	0.334
	coefficient of variation	0.243	0.301	0.340	0.610
N-Prn	maximum error	0.334	0.343	0.365	0.551
	standard error	0.059	0.084	0.131	0.098
	standard deviation	0.188	0.265	0.293	0.310
	coefficient of variation	0.437	0.620	0.688	0.728
Sn-Pg	maximum error	0.200	0.411	0.259	0.349
	standard error	0.039	0.072	0.097	0.069
	standard deviation	0.124	0.227	0.216	0.218
	coefficient of variation	0.219	0.400	0.381	0.384
N-Pg	maximum error	0.336	0.532	0.280	0.423
	standard error	0.081	0.111	0.105	0.087
	standard deviation	0.257	0.351	0.236	0.275
	coefficient of variation	0.244	0.333	0.224	0.261
Glab-Ch(r)	maximum error	0.236	0.258	0.314	0.314
	standard error	0.043	0.054	0.102	0.060
	standard deviation	0.135	0.170	0.229	0.189
	coefficient of variation	0.200	0.251	0.338	0.279
Glab-Ch(l)	maximum error	0.210	0.374	0.179	0.401
	standard error	0.040	0.074	0.078	0.080
	standard deviation	0.125	0.234	0.174	0.252
	coefficient of variation	0.184	0.343	0.256	0.370
Glab-Ft(r)	maximum error	0.205	0.223	0.179	0.179
	standard error	0.034	0.046	0.066	0.032
	standard deviation	0.107	0.147	0.148	0.102
	coefficient of variation	0.163	0.224	0.225	0.155
Glab-Ft(l)	maximum error	0.123	0.246	0.214	0.221
	standard error	0.023	0.048	0.082	0.051
	standard deviation	0.071	0.153	0.183	0.160
	coefficient of variation	0.109	0.234	0.280	0.245
Zy(l)-Tr(l)	maximum error	0.138	0.286	0.246	0.437
	standard error	0.030	0.062	0.084	0.082
	standard deviation	0.096	0.197	0.188	0.259
	coefficient of variation	0.109	0.673	0.643	0.891
Zy(r)-Tr(r)	maximum error	0.242	0.405	0.460	0.620
	standard error	0.047	0.082	0.177	0.131
	standard deviation	0.149	0.258	0.396	0.413
	coefficient of variation	0.330	0.836	1.283	1.333
Ch(r)-Tr(r)	maximum error	0.176	0.386	0.286	0.670
	standard error	0.035	0.068	0.110	0.128
	standard deviation	0.112	0.217	0.247	0.404
	coefficient of variation	0.486	0.262	0.299	0.488
Ch(l)-Tr(l)	maximum error	0.198	0.303	0.496	0.890
	standard error	0.036	0.053	0.162	0.164
	standard deviation	0.114	0.167	0.363	0.520
	coefficient of variation	0.136	0.203	0.440	0.632
Ch(r)-Gn(r)	maximum error	0.153	0.220	0.239	0.326
	standard error	0.029	0.040	0.104	0.069
	standard deviation	0.092	0.127	0.232	0.220
	coefficient of variation	0.139	0.236	0.447	0.423
Ch(l)-Gn(l)	maximum error	0.139	0.231	0.165	0.236
	standard error	0.031	0.049	0.054	0.047
	standard deviation	0.097	0.154	0.121	0.147
	coefficient of variation	0.187	0.296	0.224	0.273

**Table 3:** Mean differences with 95% confidence intervals between linear measurements performed on 3D images taken in the reference position with those obtained on acquisitions with yaw, roll, and pitch angulations. When the TOST p-value was less than 5% (*p* < 0.05) we rejected the null hypothesis of nonequivalence and concluded that the measurements were equivalent.

Measurements	Yaw		Roll		Pitch	
	Mean Difference (95% CI)	TOST *p*-Value	Mean Difference (95% CI)	TOST *p*-Value	Mean Difference (95% CI)	TOST *p*-Value
Chel(r)-Chel(l)	54.691 (52.519; 55.917)	<0.001	54.882 (53.158; 55.398)	<0.001	54.873 (53.144; 55.412)	<0.001
N-Prn	42.605 (40.107; 44.661)	<0.001	42.820 (39.941; 44.423)	0.032	42.755 (40.309; 44.055)	0.002
Sn-Pg	56.868 (55.449; 57.703)	<0.001	56.783 (55.504; 57.496)	<0.001	56.739 (55.543; 57.425)	<0.001
N-Pg	105.308 (104.394; 105.758)	<0.001	105.187 (103.996; 105.605)	<0.001	105.162 (104.187; 105.444)	<0.001
Glab-Ch(r)	67.644 (67.144; 67.802)	<0.001	67.704 (66.289; 68.172)	<0.001	67.693 (66.041; 68.029)	<0.001
Glab-Ch(l)	68.312 (66.555; 69.131)	0.006	68.062 (66.490; 68.688)	<0.001	68.074 (66.866; 68.663)	<0.001
Glab-Ft(r)	65.653 (65.747; 65.747)	<0.001	65.689 (64.768; 65.998)	<0.001	65.677 (64.851; 66.031)	<0.001
Glab-Ft(l)	65.601 (64.714; 66.012)	0.006	65.325 (63.514; 66.086)	<0.001	65.346 (63.750; 66.035)	<0.001
Zy(l)-Tr(l)	28.972 (26.219; 31.047)	<0.001	28.888 (25.126; 31.632)	<0.001	28.761 (22.139; 33.940)	<0.001
Zy(r)-Tr(r)	30.965 (26.7521; 34.341)	0.004	30.813 (25.609; 33.925)	<0.001	30.814 (26.951; 33.827)	<0.001
Ch(r)-Tr(r)	82.730 (80.600; 83.858)	0.033	82.578 (80.944; 83.062)	<0.001	82.439 (81.390; 83.014)	<0.001
Ch(l)-Tr(l)	82.154 (78.962; 83.156)	<0.001	82.169 (80.345; 82.736)	<0.001	82.026 (79.201; 82.602)	<0.001
Ch(r)-Gn(r)	53.961 (52.814; 54.712)	<0.001	54.012 (51.262; 54.785)	0.002	54.032 (53.084; 54.522)	<0.001
Ch(l)-Gn(l)	51.923 (50.621; 52.891)	<0.001	51.937 (50.835; 52.511)	<0.001	51.953 (50.788; 52.558)	<0.001

## Data Availability

The data presented in this study are available on request from the corresponding author.

## References

[1] Sforza C., De Menezes M., Ferrario V.F. (2013). Soft and Hard Tissue Facial Anthropometry in Three Dimensions: What’s New. J. Anthropol. Sci..

[2] Smeets D., Claes P., Vandermeulen D., Clement J.G. (2010). Objective 3D face recognition: Evolution, approaches and challenges. Forensic Sci. Int..

[3] Blaszczyk M., Jabbar R., Szmyd B., Radek M. (2021). 3D Printing of Rapid, Low-Cost and Patient-Specific Models of Brain Vasculature for Use in Preoperative Planning in Clipping of Intracranial Aneurysms. J. Clin. Med..

[4] Cassi D., Battistoni G., Magnifico M., Di Blasio C., Pedrazzi G., Di Blasio A. (2019). Three-dimensional evaluation of facial asymmetry in patients with hemifacial microsomia using stereophotogrammetry. J. Cranio-Maxillofac. Surg..

[5] Di Blasio A., Di Blasio C., Pedrazzi G., Cassi D., Magnifico M., Manfredi E., Gandolfini M. (2017). Combined Photographic and Ultraso-nographic Measurement of the ANB Angle: A Pilot Study. Oral Radiol..

[6] Hallac R.R., Feng J., Kane A.A., Seaward J.R. (2017). Dynamic facial asymmetry in patients with repaired cleft lip using 4D imaging (video stereophotogrammetry). J. Cranio-Maxillofac. Surg..

[7] Lauren M., McIntyre F. (2014). A New 4-Dimensional Imaging System for Jaw Tracking. Int. J. Comput. Dent..

[8] Lauren M., McIntyre F. (2013). 4D Clinical Imaging for Dynamic CAD. Int. J. Dent..

[9] Metzger T.E., Kula K.S., Eckert G.J., Ghoneima A.A. (2013). Orthodontic soft-tissue parameters: A comparison of cone-beam computed tomography and the 3dMD imaging system. Am. J. Orthod. Dentofac. Orthop..

[10] Plooij J.M., Maal T.J., Haers P., Borstlap W.A., Kuijpers-Jagtman A.M., Bergè S.J. (2011). Digital Three-Dimensional Image Fusion Processes for Planning and Evaluating Orthodontics and Orthognathic Surgery. A Systematic Review. Int. J. Oral Maxillofac. Surg..

[11] Kau C.H., Richmond S., Incrapera A., English J., Xia J.J. (2007). Three-dimensional surface acquisition systems for the study of facial morphology and their application to maxillofacial surgery. Int. J. Med Robot. Comput. Assist. Surg..

[12] Weinberg S.M., Scott N.M., Neiswanger K., Brandon C.A., Marazita M.L. (2004). Digital Three-Dimensional Photogrammetry: Evaluation of Anthropometric Precision and Accuracy Using a Genex 3D Camera System. Cleft Palate-Craniofac. J..

[13] Luebbers H.-T., Messmer P., Obwegeser J.A., Zwahlen R.A., Kikinis R., Graetz K.W., Matthews F. (2008). Comparison of different registration methods for surgical navigation in cranio-maxillofacial surgery. J. Cranio-Maxillofac. Surg..

[14] Marmulla R., Mühling J., Eggers G., Hassfeld S. (2005). Markerless patient registration. A new technique for image-guided surgery of the lateral base of the skull. HNO.

[15] (2018). Jamovi Project, Version 0.9, Computer Software. https://www.jamovi.org.

[16] Abramson J.H. (2011). WINPEPI updated: Computer programs for epidemiologists, and their teaching potential. Epidemiol. Perspect. Innov..

[17] Lung K.R., Gorko M.A., Llewelyn J., Wiggins N. (2003). Statistical Method for the Determination of Equivalence of Automated Test Procedures. J. Autom. Method Manag..

[18] Ialongo C. (2017). The logic of equivalence testing and its use in laboratory medicine. Biochem. Medica.

[19] Baysal A., Sahan A., Ozturk M., Uysal T. (2016). Reproducibility and Reliability of Three-Dimensional Soft Tissue Landmark Iden-tification Using Three-Dimensional Stereophotogrammetry. Angle Orthod..

[20] Cassi D., De Biase C., Tonni I., Gandolfini M., Di Blasio A., Piancino M. (2016). Natural position of the head: Review of two-dimensional and three-dimensional methods of recording. Br. J. Oral Maxillofac. Surg..

[21] Cooke M.S., Orth D. (1990). Five-year reproducibility of natural head posture: A longitudinal study. Am. J. Orthod. Dentofac. Orthop..

[22] Ferrario V., Sforza C., Germano D., Dallorca L., Miani A. (1994). Head Posture and Cephalometric Analyses: An Integrated Pho-tographic/Radiographic Technique. Am. J. Orthod. Dentofac. Orthop..

[23] Peng L., Cooke M.S. (1999). Fifteen-year reproducibility of natural head posture: A longitudinal study. Am. J. Orthod. Dentofac. Orthop..

[24] Magnifico M., Cassi D., Gandolfini M., Toffoli A., Zecca P., Di Blasio A. (2018). Orthodontics and Moebius Syndrome: An Ob-servational Study. Minerva Stomatol..

[25] Bister D., Edler R.J., Tom B.D.M., Prevost A.T. (2002). Natural head posture—Considerations of reproducibility. Eur. J. Orthod..

[26] Hughes G.N., Gateño J., English J.D., Teichgraeber J.F., Xia J.J. (2017). There is variability in our perception of the standard head orientation. Int. J. Oral Maxillofac. Surg..

[27] Lübbers H.-T., Medinger L., Kruse A., Grätz K.W., Matthews F. (2010). Precision and Accuracy of the 3dMD Photogrammetric System in Craniomaxillofacial Application. J. Craniofac. Surg..

[28] Aldridge K., Boyadjiev S.A., Capone G.T., DeLeon V.B., Richtsmeier J.T. (2005). Precision and error of three-dimensional phenotypic measures acquired from 3dMD photogrammetric images. Am. J. Med. Genet. Part A.

[29] Ayoub A., Garrahy A., Hood C., White J., Bock M., Siebert J.P., Spencer R., Ray A. (2003). Validation of a Vision-Based, Three-Dimensional Facial Imaging System. Cleft Palate-Craniofac. J..

